# Prognostic Modeling of Deleterious IDUA Mutations L238Q and P385R in Hurler Syndrome Through Molecular Dynamics Simulations

**DOI:** 10.3390/ph18060922

**Published:** 2025-06-19

**Authors:** Madhana Priya Nanda Kumar, Esakki Dharsini Selvamani, Archana Pai Panemangalore, Sidharth Kumar Nanda Kumar, Vasundra Vasudevan, Magesh Ramasamy

**Affiliations:** 1 Department of Biotechnology, Faculty of Biomedical Sciences & Technology, Sri Ramachandra Institute of Higher Education and Research (DU), Chennai 600116, India; madhanapriyan@sriramachandra.edu.in (M.P.N.K.); archanapai242@gmail.com (A.P.P.); u022401108@sriramachandra.edu.in (S.K.N.K.); u022401109@sriramachandra.edu.in (V.V.); 2 Department of Bioinformatics, Bishop Heber College, Tiruchirapalli 620017, India; esakkidharsini16555@gmail.com

**Keywords:** Mucopolysaccharidosis I, IDR, IDUA, molecular docking, deleterious variants, molecular dynamics

## Abstract

MPS I (Mucopolysaccharidosis type I) is a rare lysosomal storage disease originating from the deficiency of the enzyme alpha-L-iduronidase, encoded by the IDUA gene, which impairs the degradation of glycosaminoglycans (GAGs) and diminishes biological functioning across several organs. **Background:** Out of the eleven MPS disorders, MPS I includes three syndromes, of which the first, named Hurler syndrome, affects the most. **Methods:** Several in silico tools were used, such as ConSurf (73 variants), Mutation Assessor (69 variants), PredictSNP, MAPP, PhDSNP, Polyphen-1, Polyphen-2, SIFT, SNAP, PANTHER, MetaSNP (24 variants); Missense 3D-DB (11 variants) and AlignGVGD (eight variants) for physicochemical properties; and I-Mutant, Mupro, CUPSAT, and INPS for stability predictions (four variants). **Results:** A molecular docking study was performed for the two variants: L238Q and P385R scored −7.22 and −7.05 kcal/mol, respectively, and the native scored −7.14 kcal/mol with IDR as the ligand. Molecular dynamics anticipated how these molecules fluctuate over a period of 100 nanoseconds. **Conclusions:** Alpha-L-iduronidase enzyme has a critical role in the lysosomal degradation of glycosaminoglycans. According to the comparative analysis of the three structures by MDS, P385R had the least stability in all aspects of the plots. Our study demonstrates that the mutation significantly alters protein stability and binding efficiency with the ligands.

## 1. Introduction

Lysosomal Storage Diseases (LSDs) are very rare, hereditary metabolic conditions characterised by the inefficient functioning of lysosomal enzymes, leading to the buildup of excessive substrates [1,2]. These storage disorders are a group of conditions leading to diseased organs depending on the accumulation site that determines their varied symptoms based on their progression rate [2,3]. Mucopolysaccharidosis (MPS) falls under these LSDs of the 11 MPS presently identified, of which MPS I is the standard type caused by the mutation of the IDUA gene [4,5]. The IDUA gene, which codes for the lysosomal enzyme alpha L-iduronidase, breaks down dermatan sulphate (DS) and heparin sulphate (HS) in cells by hydrolyzing unsulphated alpha-L-iduronic cid in glycosaminoglycan (GAG) [5]. Because it creates a more flexible chain with higher binding potentials and controls numerous cellular functions like movement, proliferation, differentiating, angiography, and the regulation of cytokine/growth factor activities, iduronic acid in chondroitin/dermatan sulphate alters the characteristics of the polysaccharides [6,7]. A lysosome deficit brought on by a mutation in the glycosidase enzyme prevents glycosaminoglycans from breaking down sequentially. This causes chronic and progressive dysfunction of cells, tissues, and organs, as well as lysosomal accumulation and increased urine excretion of partially deteriorated dermatan sulphate as well as heparan sulphate.

Hurler syndrome, Hurler–Scheie syndrome, and Scheie syndrome are the three types of MPS I that are distinguished by the Symptom Severity Scale. The severe type of Hurler syndrome, which affects 1 in 100,000 infants, is identified by the usual facial features, abnormal hair growth, and total loss of enzyme activity that leads to the buildup of GAG sulphate in the lysosomes [8]. The condition then progresses to severe symptoms affecting several organs and causing cognitive developmental delay, heart valve-related issues, joint and vision problems, incorrect bone formation, and difficulty breathing due to their flat nasal bridge, causing the patients to have a short life expectancy of only 10 years [9]. As the most dangerous of the three, it causes macrocephaly, hydrocephaly, hepatosplenomegaly, cardiomyopathy, sleep apnea, carpal tunnel syndrome, corneal clouding, and even death [10,11]. The limited speech ability in MPS-1-H patients may be due to the enlarged tongue and hearing loss.

Diagnosing it earlier can prolong their shortened life expectancy with approved treatments like Enzyme Replacement Therapy (ERT) using the Aldurazyme enzyme via recombinant DNA technology [12,13,14,15]. Another therapeutic option for infants with Hurler patients under the age of two and a half that may help with the clinical symptoms is transplantation of hematopoietic stem cells (HSCT) [11,16]. The combined treatment shows higher levels of the IDUA gene in animals, displaying a synergistic effect than individual treatments [12]. Due to their different medical complications, the production of personalised medications is the need of the hour. Treatments specifically tailored to their physical defects due to their various medical issues could prove highly efficient and beneficial to the individual [2].

One useful strategy for cutting down on diagnostic delay is a mutational analysis of MPS I in neonates. Clinical research and publications have found at least 199 variants in the IDUA gene, including nonsense and missense mutations, insertions, deletions, and rearrangements. The in silico study of the SNPs within the IDUA gene greatly exposed the potential negative impacts of the nsSNPs on the protein. Researchers have employed a variety of approaches to boost confidence in detecting harmful mutations when prediction results vary between methods.

By studying these variations under controlled conditions, mutational analysis can help identify unknown mutations and disease precursors [17]. Several computational tools are to be used to study the effects of the mutation [18]. To streamline the critical variants based on their deleterious nature, prediction tools like ConSurf for evolutionary analysis, PredictSNP for pathogenicity prediction, I-Mutant 2.0 SEQ, and MUpro for stability prediction were utilised [19]. Engaging in research that assesses the interaction of drugs with the target protein’s effectiveness before delving into clinical trials or wet lab experiments holds significant merit within the scientific realm. This approach offers a distinct advantage by providing precise reactivity insights while conserving valuable resources. The scrutiny of genetic variations aids in understanding how an individual’s genetic makeup can influence drug effectiveness. Employing computational methodologies and tools for structural and functional analysis can evaluate mutations in the IDUA protein. This process facilitates the identification and design of potential targets, contributing to the advancement of drug discovery efforts.

## 2. Results

### 2.1. Data Retrieval

After excluding the overlaps, a total of 101 variants were retrieved from four databases: UniProt had 42, ClinVar had 55, HGMD had 66, and DisGeNET had 157. The 101 variants were selected for further computational analysis. The PDB structure 4MJ2 with a resolution of 2.10 Å was retrieved [20,21].

### 2.2. Conservation Analysis

The variants were examined, and the calculated conservation scores ranged from 7 to 9. A conservation score of 9 comprised 40 variants; a score of 8 comprised 28 variants; and a score of 7 comprised 5 variants. Finally, 75 variants come under these conserved regions.

### 2.3. Mutation Assessor

The evolutionarily conserved patterns were used to calculate the FI score (functional impact), VC score (variant conservation), and VS score (variant specificity). After removing the low and neutral FI scores, 69 variants are chosen as medium (7–9 ConSurf scores).

### 2.4. Pathogenicity

The PredictSNP tool that detects and analyses the deleterious nature of the mutants was utilized to specify the most pathogenic variant. Each sub-tool had its prediction algorithm, which PredictSNP compiled. Of the previously selected SNPs, 25 of them showed higher detrimental characteristics. The final data generated by the sub-tools, particularly PolyPhen-1, PolyPhen-2, MAPP, PhD-SNP, SIFT, SNAP, PANTHER, and MetaSNP, were presented in Table 1.

### 2.5. Biophysical Properties Analysis

The Missense 3D was used to calculate the most damaging variants from the 24 variants evaluated for their pathogenicity. A total of 11 mutants showed detrimental properties and were subjected to further studies.

The GV and GD scores and the classes were predicted using AlignGVGD. The prediction categories (C0, C15, C25, C35, C45, C55, and C65) form a continuum, with C65 being the most likely to disrupt the activity, and C0 being the least harmful. In total, eight variants: G84R, L238Q, L238R, L346P, D349Y, P385R, L396P, and G630D belong to C65 as the results of AlignGVGD.

### 2.6. Stability

The four SNPs from I-Mutant, seven from Mupro, and f from CUPSAT showed decreased stability, while all these had favourable torsions. Eight variants from INPS were destabilizing, and the L238Q, D349Y, P385R, and L346P variants were selected based on their DDG in kcal/mol value mutually from all the stability tools.

### 2.7. SNP Effect

The information regarding the two variants was retrieved from TANGO, WALTZ, LIMBO, and FOLDX using SNPeffect 4.0. The L238Q and P385R variants showed reduced protein stability and decreased binding tendency.

### 2.8. Protein Preparation

The R3 form of the protein IDUA was used in this study. The R3 forms represent the apo-state of IDUA which is crucial for structural study without bias. In this state, the enzyme is not bound to any inhibitor or substrate. The 4MJ2 model has all the domains of IDUA while others lack full domain coverage. This form has been used as standard for studying the functions of IDUA. PyMOL 2.4 was used to generate the native structure of the protein 4MJ2 in PDB format by deleting all other chains and saving them separately, and gaps were filled with the Swiss Model tool. The mutant residues at positions 238 and 385 of the protein were changed from leucine to glutamine and from proline to arginine using mutagenesis. The mutants were also saved in PDB format for further energy minimisation processes in the SPDBV 4.1.0 viewer. The energy-minimised structure was then superimposed to analyse the differences, calculated by RMSD values in PyMOL.

### 2.9. Docking

The refined native structure with the two variants docked with the IDR and scored a binding affinity score of −7.14 kcal/mol with the native, −7.22 kcal/mol with the first variant L238Q, and −7.05 kcal/mol with the second variant P385R of Hurler syndrome.

**Figure 1:**
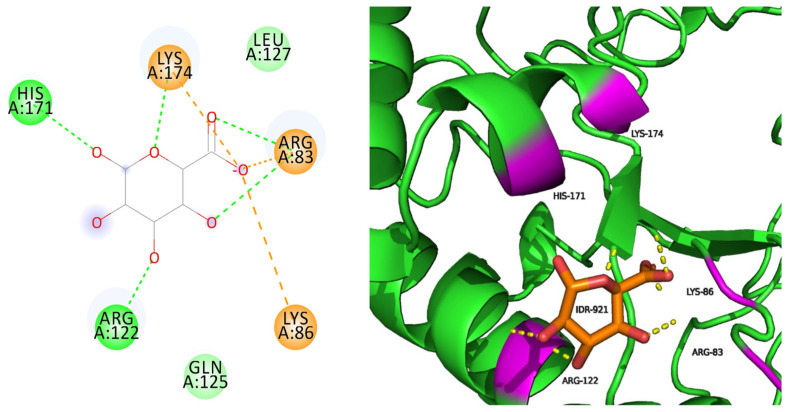
Native docked with IDR.

Native docked with IDR has five hydrogen bond interactions and three salt bridge interactions surrounded by seven amino acids, with five of them interacting (ARG83, LYS86, ARG122, HIS171, and LYS174) (Figure 1). Three salt bridge interactions have been formed, as indicated with orange lines. IDR binds efficiently with the native protein, thus influencing its stability.

**Figure 2:**
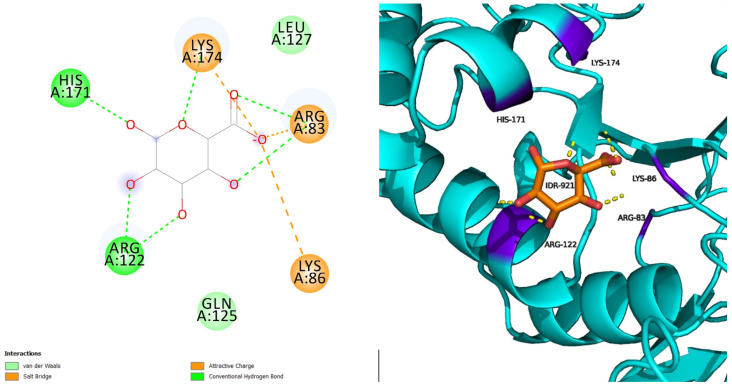
L238Q with IDR.

L238Q with IDR has five hydrogen bonds and three salt bridge interactions surrounded by seven amino acids, with the interacting residues being five (ARG83, LYS86, ARG122, HIS171, and LYS174) (Figure 2). The secondary structure of the protein remains unchanged and the interactive residues are well-positioned around the ligand.

**Figure 3:**
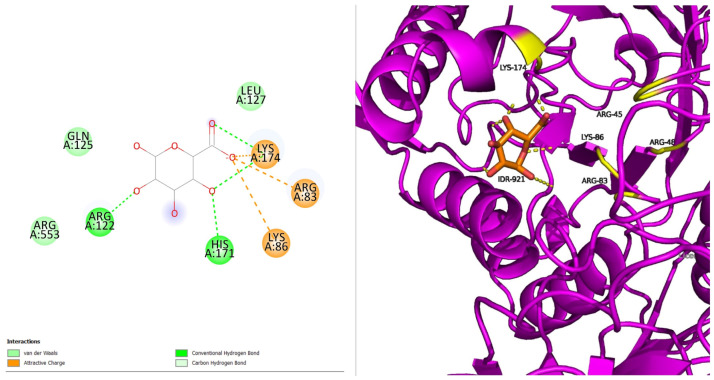
P385R with IDR.

P385R with IDR has four hydrogen bonds and three salt bridge interactions surrounded by eight amino acids, with the interacting residues being five (ARG83, LYS86, ARG122, HIS171, and LYS174) (Figure 3). The proteins’ secondary structure has minimal disruption. Additional residues suggest structural rearrangement due to the mutation. The loss in one hydrogen indicates the loss in binding affinity compared to the native.

### 2.10. Dynamics

**Figure 4:**
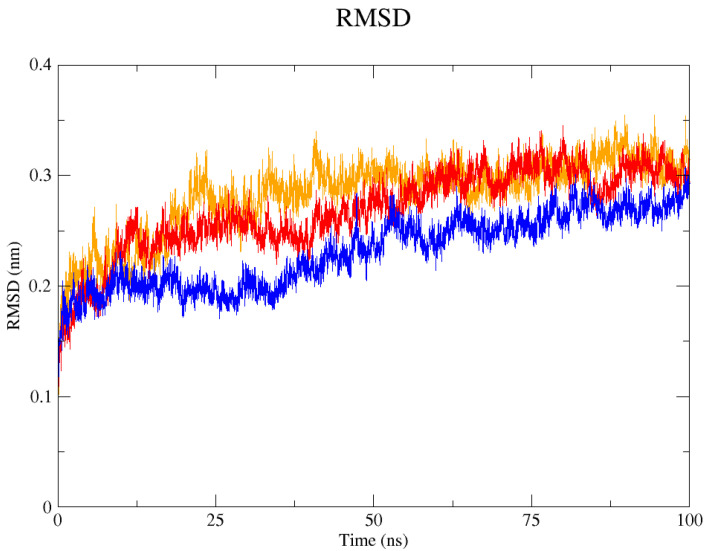
In the RMSD (Root Mean Square Deviation) graph, the native 4MJ2 is represented in blue, the mutant structures of L238Q in red and P385R in orange for 100 ns. Around 35 ns, the level of deviation increased in the two mutant proteins when compared with the native protein, and they all stabilised after 60 ns.

**Figure 5:**
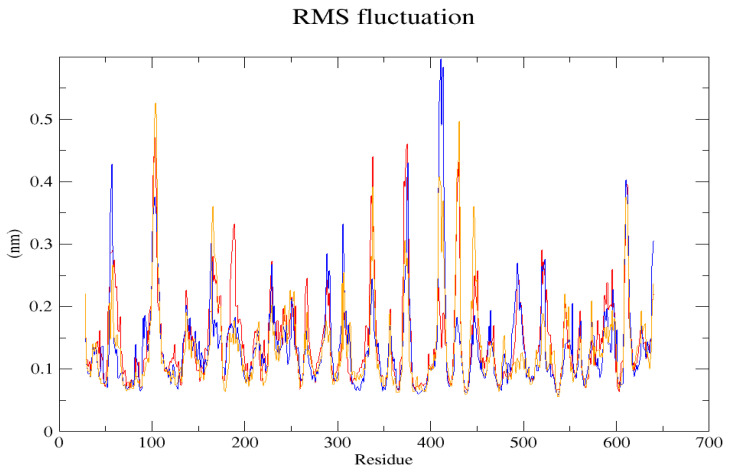
In the RMSF (Root Mean Square Fluctuation) graph, the shift between the native structure (blue) and the mutant structures L238Q (red) and P385R (orange) is visible.

**Figure 6:**
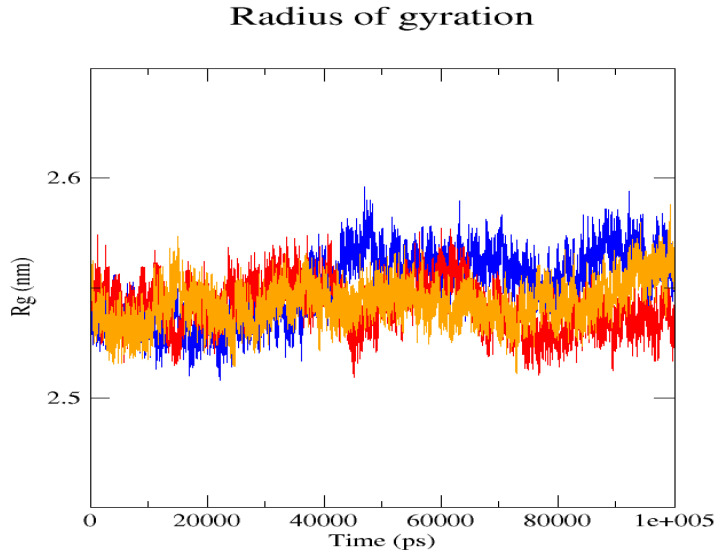
The radius of gyration (Rg) plot shows the comparison of patterns between the native structure (blue) and the two mutant L238Q (red) and P385R (orange) proteins.

**Figure 7:**
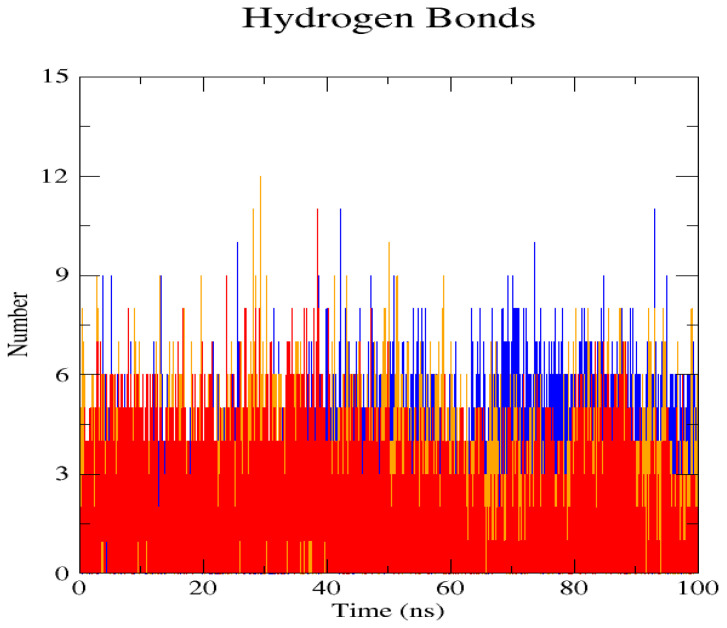
The H-bond interactions of the native structure (blue) and the two mutant proteins, L238Q (red) and P385R (orange) can be analysed in the hydrogen bond plot. The L238Q has up to 6 H-bonds overall and reaches its highest peak after 30 ns. P385R and the native have similar patterns around 3–9 numbers of H-bonds and reach their highest peak after 25 ns.

**Figure 8:**
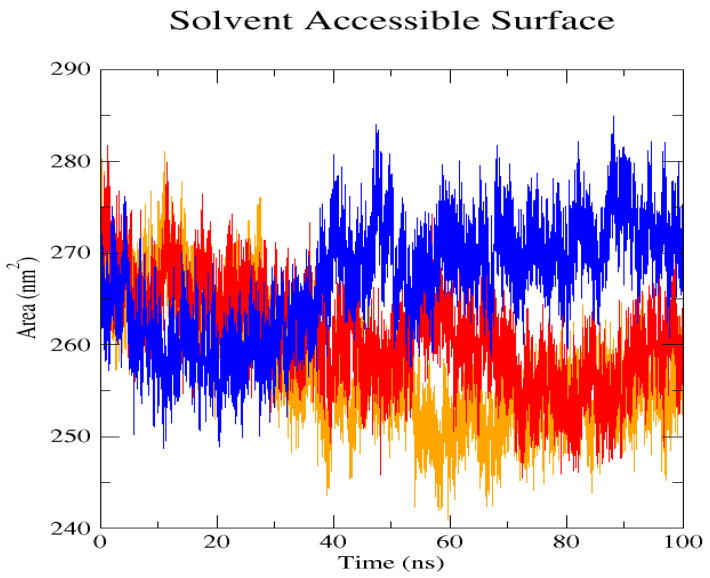
Solvent Accessible Surface Area (SASA) shows the deviation patterns between the native structure (blue) and the two mutant L238Q (red) and P385R (orange) proteins. Unlike the native structure, which deviates from 260 nm to an increasing level of 270 nm, the mutant protein structure deviated to a lower level of 275 mm to 250 nm in L238Q and P385R after 45 ns.

**Figure 9:**
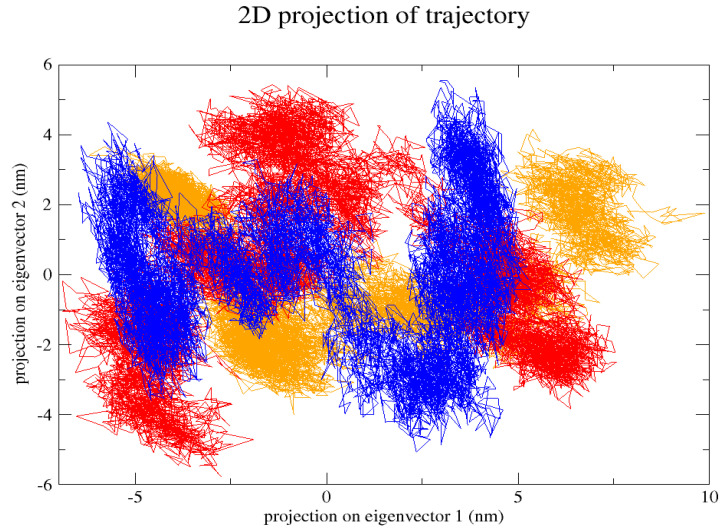
In the 2D projection of the trajectory, the native (blue) is from a −5 to 5 nm projection on eigenvector 1 and a −4 to 5 nm projection on eigenvector 2, P385R (orange) is from a −5 to 8 nm projection on eigenvector 1 and a −3 to 3 projection on eigenvector 2, and L238Q (red) is from the −5 to 5 projection on eigenvector 1 nm and from a −6 to 7 projection on eigenvector 2 nm.

The GROMACS software 4.6 was used to run molecular dynamics simulations for 100 ns on the native structure along with the two mutant structures of the variants of Hurler syndrome. The variants (L238Q and P385R) of Hurler syndrome were taken for dynamic simulations since this is the most severe case causing early death among these three syndromes. The RMSD computations are needed to determine how stable and flexible the structures are and whether they have reached the convergence level. In gmx rms format, the native structure (blue) has the fewest deviations of convergence at 0.28 nm, implying good stability when compared to the mutant structures L238Q (red) and P385R (orange), which have significant deviations (Figure 4). The RMSF assesses the structural aspects that diverge the greatest from their mean structure: the P385R mutation reaches near the native fluctuations at ~0.7 nm, the L238Q mutation at ~0.4 nm, and the native structure reaches the highest peak at ~0.9 nm (Figure 5). The radius of gyration (Rg) analysis revealed that the native protein had a slightly higher deviation of 2.58 Rg (nm) than the mutant proteins L238Q and P385R, which had deviations of 2.53 to 2.54 Rg (nm) and 2.56 Rg (nm) after 7000 ps, respectively; however, all the structures appeared to have similar Rg patterns (Figure 6). The L238Q has up to six H-bonds in the hydrogen bonding graph, with the highest peak after 30 ns. P385R and the native have similar patterns around 3–9 numbers of H-bonds and reach the highest peak after 25 ns (Figure 7). In contrast to the structure of native proteins, which starts at 260 nm and deviates to an increasing level of 270 nm after 45 ns, both the structures of the mutant proteins begin to have lower deviations of 275 nm to 250 nm in the L238Q structure, specifically the P385R in orange of 245 nm after 45 ns in the SASA plot (Figure 8). No overall structure expansion is seen in the PCA plots with the native structure in blue compared to the mutant structures L238Q in red and P385R in orange (Figure 9).

## 3. Discussion

MPS I is an autosomal recessive condition caused by a mutation in the IDUA gene on chromosome 4. In mucopolysaccharides termed dermatan sulphate and heparan sulphate, often referred to as glycosaminoglycans or GAGs, it encodes the lysosomal hydrolase alpha-L-iduronidase, which degrades unsulphated alpha-L-iduronic acid. Mucopolysaccharidosis type I (MPS I) is a lysosomal illness caused by a deficiency of the enzyme alpha-L-iduronidase (IDUA), which is necessary for the breakdown of the glycosaminoglycans, dermatan, and heparan sulphate. The disease is caused by a number of MPS I mutations that decrease alpha-L-iduronidase activity. Urinary GAG levels must be evaluated in order to identify the disorder and distinguish between the three levels of severity. Hurler syndrome was the most prevalent of the three types and had severe symptoms. Soon after birth, infants exhibit the disease’s clinical signs and abnormalities, and it progresses swiftly. The majority of patients die during the first few years of their lives.

Single-nucleotide polymorphisms (SNPs) are single variations in a specific position of the coding region of the sequence. When it substitutes the amino acids, it is a synonymous variant responsible for disease. Four databases were used to collect the disease-causing variants, including proteins with altered function and structure: UniProt, ClinVar, HGMD, and DisGeNET. The syndrome that the patients experience was linked to its most harmful variant. The database, based on the data from multiple sources, was used to examine all variants associated with MPS I disorder. From all of these databases, 219 variants were collected, and 101 variants were chosen for further analysis based on their repetition.

The initial SNP analysis included the analysis for conservation. The high scores in ConSurf from 7 to 9 indicated a better probability of conservation in the amino acid positions. A total of 75 of them proved to play a role in the causation of MPS 1. MutAssessor concise the variant list by eliminating the variants with low functional impact, bringing it down to 69 SNPs. The most detrimental mutants enhance the pathogenicity and severely impact the functioning of the enzyme. PredictSNP, an in silico tool, aided in the identification of the most pathogenic out of the list, totalling up to 25 variants. The Missense 3D analysis results on the website provide a concise and detailed structural explanation for each variant, along with a user-friendly 3D interactive display to view the wild-type and mutant types. The biophysical analysis of the protein determines the functionality post amino acid substitution. In AlignGVGD, the biochemical variation observed at each alignment position is transformed into a Grantham Variation score (GV). Subsequently, the disparity between these characteristics and those of the variant amino acid under evaluation is computed, generating a Grantham Difference score (GD). 8 SNPs belonging to the C65 group indicated the inherent hindrance of functionality in eliminating GAGs. The stability of the selected variants was determined using I-Mutant, Mupro, CUPSAT, and INPS. The delta G values of the variants compiled from other subtools were close to 1, revealing its instability in active site binding. The resultant four mutants were considered the most deleterious after SNPeffect analysis.

The energy minimisation (EM) method was computed with the help of the Swiss PDB Viewer (SPDBV) program, thereby improving the protein’s stability. Computational drug discovery methods highlight the virtual understanding of protein-ligand interaction systems. Molecular docking aids in understanding how the drug binds with the target molecule by utilising AutoDock 4.0 software and determining the best binding complex through a comparative study of the two variants and the native. When IDR docks with the native, five hydrogen bond interactions were observed between them: L238Q had five hydrogen bonds, and P385R had four hydrogen bonds. According to the binding energy calculated through the software, both the variants L238Q and P385R showed higher values than the native IDUA protein. The results imply that the mutations are detrimental and prevent effective binding of the ligand to our target protein, resulting in poor enzyme function. Both SNPs were processed further to comprehend their stability and flexibility during an enzymatic reaction.

Perspectives from MD simulations complement docking studies of these structures significantly. The RMSD computations show that the native structure was more stable than the mutant structures L238Q and P385R, which have the highest deviations. The P385R pattern reaches near the native structure, which had higher fluctuations than the L238Q, as analysed through the RMSF graph and the radius of gyration (Rg). In the Solvent Accessible Surface Area plot, the mutant structure displayed reduced deviations in the L238Q structure around 45 ns in contrast to the original protein. All the structures were between the 3 and 6 bonds on the H-bonds graph, and P385R had higher fluctuations than L238Q. However, after 45 ns, the fluctuations started to alter, presenting a higher deviation as in the SASA plot of the native protein. The trajectory files were analysed. The XVG file was plotted using the XMGRACE software 5.1. Thus, L238Q had a higher elevation than the other two patterns analysed from the MD graphs.

The substitution of Leucine with glutamine (L238Q) adds polarity to the hydrophobic region of the IDUA enzyme, which reduces the enzymes’ key ability to hydrolyze GAGs, and thus leads to the accumulation of substrates in the lysosomes. Substitution of Proline with Arginine (P385R) changes the electrostatic interaction region into a flexible region; this disrupts the backbone geometry, which in turn alters the active site configuration leading to compromised enzymatic functions.

While computational analysis offers valuable insights into the mutations they are not sufficient for developing therapeutic drugs. In the future, biochemical assays such as enzyme kinetics can be done to measure IDUA activity in both wild-type and mutant as well as Differential Scanning Colorimetry (DSC) can be used to validate the effects of mutations.

## 4. Methods

### 4.1. Protein Retrieval

The IDUA protein sequence was obtained in FASTA format from the UniProt database with Accession ID P35475 with a protein length of 653aa [20]. The protein structure was selected based on low resolution and full coverage from the RCSB PDB database [21,22].

### 4.2. Variants Data Acquisition

The missense variants were collected from UniProt, ClinVar (https://www.ncbi.nlm.nih.gov/clinvar (accessed on 26 December 2024)), HGMD (http://www.hgmd.cf.ac.uk/ac/index.php (accessed on 26 December 2024)), and DisGeNET (http://www.disgenet.org (accessed on 26 December 2024)) databases with “IDUA” and “Homosapiens” as keywords for the search [23,24]. In addition, the term, “Hurler Syndrome” was used in the DisGeNET database for specialised results [25]. The in silico tools selected for this analysis were chosen to target specific aspects of a protein which ensures a multifaceted approach towards these mutations. Using multiple tools helps in reducing false predictions by validating the obtained results across differing algorithms. Tools like AutoDock provide static insights into the interactions while MD helps in visualizing the interactions dynamically.

### 4.3. Conservational Analysis

The ConSurf tool enables the analysis of protein evolutionary conservation patterns. The difference between the ‘conserved’ regions and the ‘varied’ regions can be visualised using the scale of scores with certain colors [26]. The retrieved FASTA sequence was pasted and submitted with default parameters. The ConSurf tool represents the scales of variability (1–3), average (4–6), and conservation (7–9) regions based on the Multiple Sequence Alignment (MSA) between the family of organisms [27].

### 4.4. Mutation Assessor

The Mutation Assessor tool estimates how point mutations will affect the protein sequence’s functionality effect [26,28]. This tool was run with the variants selected based on these conservation scores to predict the functional impact of a variant [28]. The impact can be functional, which is high or medium, or “non-functional”, which is low or neutral. The input format should be the UniProt accession and its respective variant, “IDUA_HUMAN & Variant”. The option “individual scores, misc. msa info”, was chosen to get the functional impact (FI), VC, and VS scores using the conservation patterns partitioning disease variants, especially highly functional scores. The results were downloaded as a “.csv” file for each variant for future analyses.

### 4.5. Analysing the Variant Function

The PredictSNP server [29] is a pathogenicity prediction tool comprising various tools, including MAPP, SNAP, PhD-SNP [30,31], Polyphen-1, Polyphen-2, SNAP, PANTHER, and SIFT [32]. The protein sequence of IDUA was provided as input in the FASTA format along with all the mutations and, the effective amino acid substitutions were selected.

- SNAP: The reliability index (RI), which runs from 0 to 9, the binary prediction (neutral or non-neutral), and the estimated accuracy are all predicted by SNAP for each amino acid mutation [33]. If the predicted values are greater than 0.5, the mutations are classified as disease-causing variants; otherwise, they are classified as neutral mutations [30].
- PhD-SNP (Predictor of Human Deleterious Single-Nucleotide Polymorphisms): A user-friendly platform for interpreting the effect of SNVs (single-nucleotide variations) in coding and non-coding sites is the PhD-SNP online server [34,35]. The PhD-SNP is based on a decision tree, and it is connected with an SVM profile that has been developed using data from the sequence profile. If the estimated values are greater than or equal to 0.5, the mutations are categorised as deleterious variants; otherwise, they are classified as neutral mutations [36].
- MAPP: An effective bioinformatic tool that outperformed other missense variant classification algorithms by a wide margin, interpreting missense variants in a way that precisely distinguishes harmful from neutral variants [37].
- PolyPhen-1 (Polymorphism Phenotyping): Evaluates how an amino acid change affects a human protein’s structure and functionality. A Naive Bayes classifier is used to predict the detrimental effects of an amino acid alteration and the way it would affect the stability and function of the protein. PolyPhen2 (Polymorphism Phenotyping v2) uses both sequence homology and structural data.
- Sorting Intolerant from Tolerant (SIFT): https://sift.bii.a-star.edu.sg/ (accessed on 26 December 2024) is accessible. Given the homology of the sequence and the physical closeness of the alternative amino acids, an algorithm examines possible modifications in protein function. This yields both a qualitative prediction and a score. The SIFT method considers greater values as neutral changes and less than 0.05 as a variation that causes illness.
- PANTHER is well-balanced in representing the human pathogenic variants and works using a metric based on evolutionary preservation [38]. It is critical to monitor mutations that affect the protein’s stability and structure.
- Meta-SNP predictions based on mutation position achieve 79% overall accuracy, which improves disease detection [39].

### 4.6. Variant Analysis

The IDUA gene was located in the Missense3D-DB (http://missense3d.bc.ic.ac.uk:8080/ (accessed on 26 December 2024)) to identify the total count of the variants and the count of both the neutral and damaging variants. A position was given to each variant, including the residue’s wild type and mutant, for the specific prediction of whether it is damaging or neutral [40].

The result page of the missense 3D analysis predicts a concise and in-depth structural description for each variant, whether they are pathogenic, as well as a user-friendly interactive 3D viewer of the wild and mutant types [40,41].

### 4.7. Biophysical Analysis

Align-GVGD (http://agvgd.hci.utah.edu/ (accessed on 26 December 2024)) predicts the severity of missense substitutions using the Grantham deviation (GD) and Grantham Variation (GV). Align GVGD was used by selecting “run the program” and pasting the FASTA sequence. The list of substitutions was submitted for the prediction. According to the predictions of the classes C65, C55, and C15, only the C65 classes were selected [42].

### 4.8. Stability Prediction

Stability analyses have been carried out to study the effects of deleterious mutations on the protein using the following standard tools: I-Mutant 2.0, MUpro, CUPSAT, and INPS.

- I-Mutant 2.0 [43] Available: https://folding.biofold.org/i-mutant/i-mutant2.0.html (accessed on 26 December 2024) is a web server that can predict how much a mutation will alter the stability of the free energy state by pasting the sequence, position, and new residue of the variant along with the temperature and pH to predict the “sign of DDG” [44].
- MUpro investigates the effect of single amino acid changes on its stability [45]. The value indicates whether the mutation is expected to increase or decrease protein stability, with a score close to 1 indicating significant confidence in decreased stability [45,46]. Mupro was used with the help of the sequence as a query, mutation position, and original and substitute amino acids to predict the delta G value. With the PDB ID 4MJ2, we can predict mutant stability from existing PDB structures.
- CUPSAT is a program that predicts variations in protein stability caused by point mutations by employing amino acid-atom prospects and torsion angle distribution. Thermal was selected as the experimental method, and stability prediction was done with one amino acid, a residue number, and a chain ID selected.
- The INPS 3D (Impact of Non-synonymous Mutations on Protein Stability) technique is a sequence-based method for predicting the effects of nsSNPs, requiring a PDB file and the mutation file containing all the variants, along with the chain for the stability change (DDG) value in kcal/mol.

### 4.9. SNP Effect Analysis

The SNPeffect 4.0 database incorporates several predictions, including LIMBO to find out whether the mutation affects the chaperone binding tendency of the protein and FoldX to analyse whether the mutation enhances the protein’s stability [47,48]. The variants chosen from the stability predictions are then used in the SNP effect by searching the gene sequence for each variant. LIMBO was used to determine the chaperone binding tendency, and FOLDX was used to investigate protein stability and folding [49,50].

### 4.10. Structural Analysis

The protein preparation was done for the selected protein downloaded from the RCSB-PDB [51]. The SWISS-MODEL was used to fill the gaps in the protein by pasting the sequence in FASTA format to search for templates [52]. The 4MJ2 1. A structure was selected as a template to build a model and saved in PDB format.

The mutant structure was modeled using the Mutagenesis Wizard of PyMol Molecular Graphics System software 2.4 (https://pymol.org/2/ (accessed on 26 December 2024)). The target amino acid position from the native IDUA protein was selected for mutagenesis, followed by the use of the Mutagenesis tool to substitute it with the mutated amino acid. The molecule was exported and saved in PDB format. The same procedure was followed for each residue individually.

### 4.11. Energy Minimisation

The energy minimisation step was done for both the saved mutant structures, along the native protein structures in SwissPDBViewer (SPDBV) [53]. The native and variant structures were imported individually, and every residue was selected. Energy minimisation is conducted using a limited application of the GROMOS96 force field. This process is aimed at rectifying geometric irregularities by repositioning atoms to alleviate internal constraints. The structures’ minimal energies were recorded and saved in PDB format for docking purposes.

### 4.12. Protein and Ligand Preparation

Polar areas were added, Gasteiger charges were calculated, the AD4 type was assigned, and the proteins were saved in the form of PDB. To show that hydrogen bonds are present, polar areas are added to electronegative atoms in the protein, such as oxygen and nitrogen. To conveniently provide partial charges to the protein, Kolmann charges are used. The Gasteiger charge computation method was made possible by the partial charges, which relied on the normalisation of orbital electronegativity. The computation focuses on atom connection and only takes into account the topology of the molecule. Two methods were used for attaching electrophilic libraries to their target proteins: the flexible side-chain attachment method, which was first developed for modeling covalent docking in AD4, and the reactive docking strategy, which integrates ligand reactive data into a docking simulation. The macromolecule was saved in PDBQT format after being opened under the grid section. The file type was changed in order to prepare the ligand. Before saving the file in PDBQT format, commands such as “choose root”, “detect root”, and then “choose torsions” were executed in the torsion tree.

### 4.13. Docking

Intrinsically Disordered Region (IDR) was taken as a ligand for our work because these are protein segments that lack a fixed three-dimensional structure under various physiological conditions, which helps with the interactions with multiple targets and more flexibility. While analyzing proteins like IDUA, this adaptability is important as the protein-ligand interactions are critical for enzymatic activity and stability. The Prepared IDUA macromolecule and the prepared ligand were opened in the PDBQT format. The “Select from string” was selected to add the “molecule list”, “chain list”, and the active site residues of the protein, which were predicted using the CASTp 3.0 web server [54]. After the grid box values were set in the grid section, the file was saved in GPF format to run the auto grid.

The prepared macromolecule was docked with the “set rigid” option. The “genetic algorithm” docking parameter and AutoDock’s default settings were applied. The following parameters were used to choose Lamarckian GA in the output: a population of 150, an energy assessment of 2,500,000, generations of 27,000, a mutation rate of 0.02, and a boundary level of 0.8. The file was saved in DPF format, and AutoDock was launched [55]. The DLG file was opened in the Analyses section to analyse the interactions. The conformations can be ‘play ranked by energy’, and the binding affinity was noted. Then, the saved PDBQT file was opened and saved in PDB format for further analysis of the interactions between the protein and the ligand [56].

### 4.14. Dynamics

GROMACS software was used to perform the complex structures of the mutant and native [53,55,57]. To support the GROMACS software, the PDB file format was converted to the GRO format using a command on Ubuntu. Then, the GROMOS force field was used for the topology building of the protein and the ligand [58].

Since it involves both complexes of the protein and ligand, the ‘dodecahedron box’ was used to accommodate the structure in the centre. The protein structure was placed 1 nm from the outer layer of the box, which is filled with the solvent SPC (Simple Point Charge). Before the process of system neutralisation, all the molecular dynamics parameter files (MDP) were downloaded and stored in a separate file folder for Gromacs on the desktop. The neutralisation was done by adding Na+ and Cl− ions and also the energy minimisation (em) using the commands [58,59]. The temperature and pressure (NVT and NPT) were further stabilised to replicate/relate to the human body. MD simulation was initiated, and the trajectory files were analysed for 100 ns done previously [60].

## 5. Conclusions

In the present study, we retrieved 101 variants with the help of four databases, UniProt, ClinVar, HGMD, and DisGeNET, for the Hurler syndrome since it is the most severe one among the three syndromes. Several computational tools like ConSurf, Mutation Assessor, PredictSNP, MAPP, PhDSNP, Polyphen-1, Polyphen-2, SIFT, SNAP, PANTHER, MetaSNP, Missense 3D-DB, AlignGVGD, I-Mutant, Mupro, CUPSAT, and INPS were used to filter out only the deleterious variants by checking for conservation, pathogenicity, and other physicochemical properties. Then, SNPeffect 4.0 is used to predict their chaperone binding and stability levels using LIMBO and FOLDX. The two most deleterious variants, L238Q and P385R, found in the analyses were docked with IDR using the AutoDock software. The variant with a score of −7.22 kcal/mol (L238Q) had the best binding affinity. The same variant showed the best results when performing the RMSD, RMSF, radius of gyration (Rg), hydrogen bond, and SASA plots in the molecular dynamics simulations using the GROMACS software. To enhance the prospects of drug development, future studies should focus on the experimental validation of the ligands either through kinetic assays or by other techniques, such as Differential Scanning Calorimetry (DSC). These approaches can help to better understand the functional impact of the mutations and their role as therapeutic targets.

## Figures and Tables

**Table 1:** Pathogenicity analysis of IDUA variants.

PredictSNP Sub Tools	Count Percentage	Variants Count
PolyPhen-1	88%	58
PolyPhen-2	100%	66
MAPP	100%	58
PhD-SNP	73%	48
SIFT	92%	61
SNAP	86%	57
PANTHER	65%	43
MetaSNP	79%	52

## Data Availability

All data generated or analysed during this study are included in this published article.

## Abbreviations

AlignGVGD	Align Grantham Variation Grantham Deviation
CUPSAT	Cologne University Protein Stability Analysis Tool
DDG	Change in the change in Gibbs free energy (Double changes intended)
GAG	Glycosaminoglycans
GROMACS	Groningen Machine for Chemical Simulations
MD	Molecular Dynamics
MMPBSA	Molecular mechanics Poisson–Boltzmann surface area
Rg	Radius of Gyration
RI	Reliable Index
RMSD	Root Mean Square Deviation
RMSF	Root Mean Square Fluctuation
SASA	Solvent Accessible Surface Area
SNAP	Screening for Non-Acceptable Polymorphisms
SPDBV	Swiss PDB Viewer
